# Nrf2 as a master regulator of tissue damage control and disease tolerance to infection

**DOI:** 10.1042/BST20150054

**Published:** 2015-08-03

**Authors:** Miguel P. Soares, Ana M. Ribeiro

**Affiliations:** * Instituto Gulbenkian de Ciência, Rua da Quinta Grande, 62, 6, 2780-156 Oeiras, Portugal

**Keywords:** disease tolerance, infection, Nrf2, oxidative stress, tissue damage control

## Abstract

Damage control refers to those actions made towards minimizing damage or loss. Depending on the context, these can range from emergency procedures dealing with the sinking of a ship or to a surgery dealing with severe trauma or even to an imaginary company in Marvel comics, which repairs damaged property arising from conflicts between super heroes and villains. In the context of host microbe interactions, tissue damage control refers to an adaptive response that limits the extent of tissue damage associated with infection. Tissue damage control can limit the severity of infectious diseases without interfering with pathogen burden, conferring disease tolerance to infection. This contrasts with immune-driven resistance mechanisms, which although essential to protect the host from infection, can impose tissue damage to host parenchyma tissues. This damaging effect is countered by stress responses that confer tissue damage control and disease tolerance to infection. Here we discuss how the stress response regulated by the transcription factor nuclear factor-erythroid 2-related factor 2 (Nrf2) acts in such a manner.

## Introduction

Resistance to infection defines a defence strategy that limits host disease severity via immune driven mechanisms that target pathogens for expulsion, containment or killing. Disease tolerance defines a distinct defence strategy that limits host disease severity without however, targeting pathogens [1–3]. Described originally in plants [4], disease tolerance is operational in flies [5–7] and mammals, including in mice [8,9] as well as in humans [10]. The term disease tolerance is used hereby to refer explicitly to the defence strategy defined originally in the plant literature [4,11], which limits host ‘damage to functions and structures’ [4] imposed by infection, without interfering with host pathogen load [4,11].

Disease tolerance is regulated by a number of evolutionarily conserved stress and/or damage responses. These confer tissue damage control, i.e. prevent ‘damage to functions and structures’ imposed by infection [4,12]. Presumably, stress and/or damage responses evolved from ancestral forms of life where they provided cellular adaptation to environmental changes [13]. Much like resistance mechanisms, these adaptive responses evolved, most probably, under the selective pressure imposed by host microbe interactions.

Resistance mechanisms can elicit, *per se*, varying levels of cellular stress and damage to the host parenchyma, as illustrated for innate immune responses associated with the production of reactive oxygen species (ROS) and/or reactive nitrogen species (RNS). This is coupled to a countervailing oxidative stress response regulated by nuclear factor-erythroid 2-related factor 2 (Nrf2), a member of the cap'n'collar basic leucine zipper family transcription factor characterized structurally by the presence of Nrf2–ECH homology domains [14]. Other members of this family include NF–E2 p45, Nrf1 and Nrf3 [14].

## Mechanisms regulating Nrf2 activation in the context of infection

**Figure 1:**
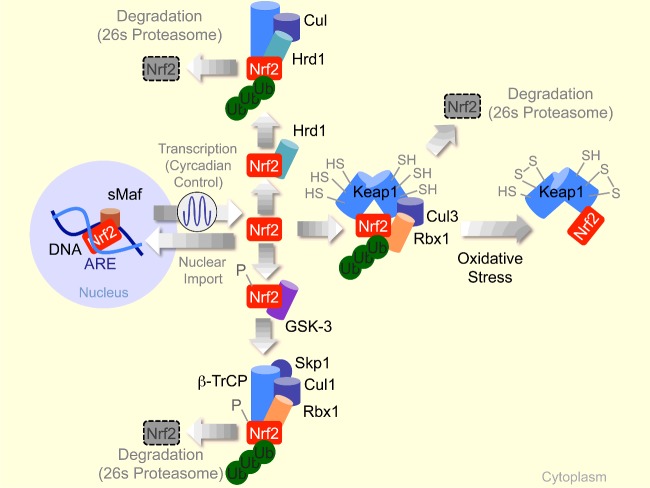
Control of Nrf2 activation by different E3 ubiquitin ligase complexes Acronyms are defined throughout the text. When no longer targeted for degradation by E3 ubiquitin ligase complexes, Nrf2 activity is controlled mainly by its rate of transcription, with newly transcribed Nrf2 regulating gene expression. It is the Keap1–Cul3–Rbx1, Hrd1 E3 ubiquitin ligase and SCF^β-TrCP^ complexes, however that underlie the stress responsive nature of Nrf2 activity.

**Figure 2:**
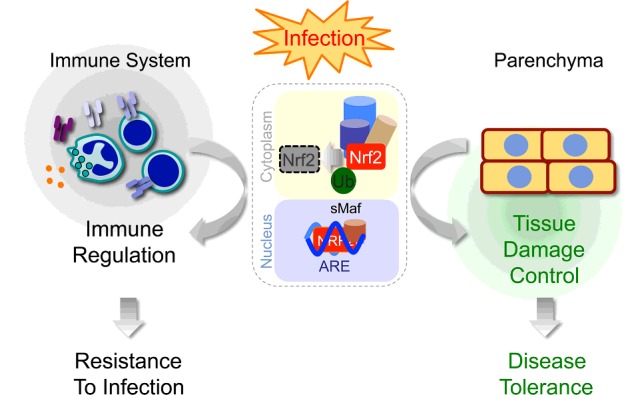
Outcomes of Nrf2 activation Upon infection, activation of Nrf2 in different cellular components of the immune system acts in an immunoregulatory manner, which modulates resistance to infection. Activation of Nrf2 in parenchyma tissues provides tissue damage control and disease tolerance to infection. Control of Nrf2 activation is illustrated in the context of a generic E3 ubiquitin ligase complex, detailed under Figure 2.

Engagement of pattern recognition receptors (PRRs) by pathogen-associated molecular patterns (PAMP) activates Nrf2 in innate immune cells such as monocytes/macrophages (Mø). For example, lipopolysaccharide (LPS) recognition by toll-like receptor 4 (TLR4) triggers the transcription/expression of the inducible form of nitric oxide synthase (iNOS/NOS2), via a mechanism involving the adaptor molecule Myd88 (myeloid differentiation primary response gene 88) and the transcription factor nuclear factor kappa B (NF-κB) [15]. The TLR4–MyD88–NF-κB signal transduction pathway also triggers the transcription/expression of the phagocytic NADPH oxidase (NOX2/gp91^phox^) [16], which generates intracellular superoxide (O_2_^●−^). The NO generated by iNOS reacts with O_2_^●−^ and produces peroxinitrate (ONNO^−^) anions, which targets several thiol-based (S-H) redox systems, including reactive cysteines in the Kelch-like ECH-associated protein 1 (Keap1) [13,17,18] (Figure 1). Keap1 is an adaptor for the cullin (Cul)3–RING (really interesting new gene)-box protein (Rbx)1 ubiquitin ligase complex, which targets Nrf2 constitutively for proteolytic degradation by the 26s proteasome [13]. Under oxidative stress, some of the reactive cysteines of Keap1, i.e. Cys^151^ are targeted by ONNO^−^, generating thiol oxidation products and ultimately forming disulfide bonds [19]. These alter the tertiary structure of Keap1, inhibiting its ubiquitin ligase activity and Nrf2 degradation [13,17,18]. The newly transcribed Nrf2 undergoes nuclear translocation and binds to small musculoaponeurotic fibrosarcoma (sMaf) transcription factors, including MafF, MafG and MafK [14], driving the transcription of Nrf2-responsive genes containing DNA antioxidant responsive elements (AREs) in their promoter [13] (Figure 1). In addition, NF-κB also acts directly on the Nrf2 promoter to induce Nrf2 transcription [13], presumably required to sustain Nrf2-dependent gene expression (Figure 2).

It is now clear that other E3 ubiquitin ligase complexes contribute to integrate Nrf2 activation within different forms of cellular stress [13]. These include the Skp1 (S-phase kinase-associated protein 1)–Cul1–F-box (SCF)–β-transducin repeats-containing proteins (β-TrCP) complex (SCF^β-TrCP^) [20], which recognizes the Neh6 (Nrf2-ECH homology 6) domain of Nrf2 when phophorylated by the glycogen synthase kinase 3 (GSK3) [20]. Presumably, Nrf2 phosphorylation at the Neh6 domain allows for coupling of different forms of stress sensed by GSK3 with Nrf2 ubiquitination by the SCF^β-TrCP^ complex and its degradation by the 26s proteasome [13,20] (Figure 1). The HMG (high mobility group)-coA reductase degradation 1 (Hrd1) E3 ubiquitin–protein ligase involved in endoplasmic reticulum-associated protein degradation (ERAD) also controls Nrf2 activation [21]. Hrd1 targets the Nhe4–5 domain of Nrf2 for ubiquitination and degradation by the 26s proteasome [21] (Figure 1). How Hrd1 acts in the context of other components of the endoplasmic reticulum stress response, such as the protein kinase RNA-like ER kinase 1 (PERK1) [22], to regulate Nrf2 is not clear.

It is worth noting that Nrf2 activity is controlled to a large extent by its rate of transcription/expression (Figure 1). This is regulated by several transcription factors including NF-κB and Nrf2 itself, as well as clock components that impose a circadian control to Nrf2 activity [23] (Figure 1).

## Nrf2 and resistance to infection

Perhaps the best demonstration that Nrf2 modulates host resistance to infection is provided by the observation that deletion of the *Nrf2* allele in mice enhances resistance to Marburg virus infection [24]. This effect is mediated by the Marburg virus encoded VP24 protein, which binds the Kelch domain of Keap1 and inhibits the ubiquitin ligase activity of the Keap1–Cul3–Rbx1 complex, hence inducing Nrf2 activation [24,25]. Several other observations are consistent with the notion that viruses induce host Nrf2 activation *in vitro*, as suggested for Kaposi's sarcoma-associated herpes virus [26], as well as for Influenza [27,28] and dengue [29] viruses. However, the pathophysiologic relevance of these observations remains to be elucidated. Conversely, other viruses such as hepatitis C virus, down-regulate Nrf2 activation via a mechanism impairing its nuclear import through delocalization of sMaf proteins [30]. The impact of this phenomenon to the outcome of hepatitis C virus infection is also not clear.

Intracellular bacteria also modulate Nrf2 activation, as demonstrated for *Salmonella typhimurium* infection in Mø [31]. Activation of Nrf2 enforces the transcription/expression of Ferroportin-1, an iron exporter that decreases iron cellular content [31]. This limits *Salmonella* access to iron, restraining the proliferation of this intracellular pathogen [31]. Whether Nrf2 acts under pathophysiologic conditions to promote resistance to *Salmonella* infection is likely, but this remains to be formally demonstrated [31]. Pharmacologic activation of Nrf2 by sulforaphane promotes resistance to *Pseudomonas aeruginosa* [32] as well as to *Plasmodium* infection in mice [33].

## Nrf2 in tissue damage control and disease tolerance

The Nrf2 signal transduction pathway also confers tissue damage control and disease tolerance to systemic infections. One of the mechanisms via which this occurs involves the establishment of a functional cross-talk between the gasotransmitters NO and CO, as illustrated for *Plasmodium* infection [34,35]. When applied pharmacologically, both NO [35–37] and CO [34,38,39] can suppress the development of experimental cerebral malaria in mice, a lethal form of severe malaria that resembles, in many aspects, human cerebral malaria [40]. This protective effect acts via Nrf2 activation by NO [41], presumably through a mechanism targeting Keap1 at Cys^151^ [13,42], but this has not been established experimentally. Nrf2 activation induces HO-1 (heme oxygenase-1) expression and the production of CO, via haeme catabolism by HO-1, which acts ultimately as the gasotransmitter suppressing the onset of experimental cerebral malaria [41]. This occurs via a mechanism involving the binding of CO to the prosthetic haeme group of cell free haemoglobin generated during the blood stage of *Plasmodium* infection, thus preventing haeme from participating in the pathogenesis of experimental cerebral malaria [34,38,39,41]. The protective effect exerted by the NO->Nrf2->HO-1->CO signal transduction pathway is not associated with modulation of host pathogen load, suggesting that the cross-talk established between these two gasotransmitters confers disease tolerance to *Plasmodium* infection via a mechanism regulated by Nrf2 [11,41].

Presumably, the mechanism via which Nrf2 confers tissue damage control and disease tolerance to malaria also involves the expression of Nrf2-responsive genes regulating haeme/iron metabolism [43]. These include the iron storage protein Ferritin H chain (FtH) [44,45], which can confer *per se* tissue damage control and disease tolerance to malaria in mice [10].

There is further evidence that argues strongly for a central role of the Nrf2 signal transduction pathway in the establishment of disease tolerance to *Plasmodium* infection. In a similar manner to humans carrying hemizygous sickle mutations in the β-chain of haemoglobin, transgenic sickle haemoglobin mice are protected from cerebral malaria [38]. This protective effect is exerted irrespectively of parasite load, revealing that sickle haemoglobin can confer disease tolerance to *Plasmodium* infection [11,38]. Sickle haemoglobin induces the expression of HO-1 through a mechanism regulated by Nrf2 and leading to the production of CO, which confers tissue damage control and disease tolerance to malaria [38,39]. Whether this mechanism explains how sickle haemoglobin protects humans from malaria remains to be established but is likely to be the case.

It is probable that a similar mechanism underlies the protective effect exerted by other chronic haemolytic conditions against malaria, including haemoglobin C [46,47], glucose 6 phosphate dihydrogenase (G6PD) deficiency in males [48], β- or α-thalassemia [47] as well as mutations underlying red blood cell cytoskeleton or membrane protein defects [49]. Presumably, the protective effect associated with these mutations is mediated via different mechanisms that converge at the level of Nrf2 activation. Therefore it is possible that sickle haemoglobin and probably these other red blood cell mutations co-evolved with the Nrf2 signal transduction pathway to limit disease severity driven by these mutations while conferring protection against malaria, such as illustrated for the sickle haemoglobin [38].

There is also circumstantial evidence to suggest that Nrf2 confers disease tolerance to systemic infections, other than malaria. Namely, Nrf2 is protective against endotoxic shock [50], severe sepsis triggered by polymicrobial infection [50] and lung injury induced by *Staphylococcus aureus* infection [51] in mice. These salutary effects have been associated mainly with immunoregulation but there is no clear evidence whether Nrf2 modulates pathogen load in these specific experimental settings [50]. Our own data confirms that Nrf2 activation prevents the lethal outcome of polymicrobial sepsis in mice, without however interfering with pathogen load (Weis, S., Ribeiro, A. and Soares, M.P., unpublished observation). This suggests that Nrf2 can confer disease tolerance to infection, presumably acting as an immunoregulatory transcription factor in innate immune cells and/or parenchyma cells to provide tissue damage control, although this remains to be fully established.

## Mechanisms underlying the protective effect of Nrf2 against infection

There is a general consensus that Nrf2 is protective against systemic infections, via a mechanism targeting NF-κB and modulating pro-inflammatory gene expression in Mø [50,52] (Figure 2). However, Nrf2 activation is required to sustain interleukin (IL)-1β secretion in Mø, via a mechanism involving NLRP3 (NACHT, LRR and PYD domains-containing protein 3) driven caspase 1 activation, an essential step in the processing of pro-IL-1β towards IL-1β secretion [53]. This would argue that Nrf2 promotes, rather than restrains, inflammation. Moreover, Nrf2 induces the expression of the activating transcription factor 3 (ATF3) in Mø, an IL-6 repressor that is protective against LPS but highly deleterious against bacterial infection [54]. This suggests that Nrf2 can also act in a deleterious manner in the context of systemic bacterial infections (Figure 2).

Oxidative stress can trigger parenchyma cells to undergo regulated necrosis [55], leading to tissue damage and organ dysfunction, eventually compromising disease tolerance to infection [12]. Therefore, host protective mechanisms that prevent parenchyma cells from undergoing regulated necrosis, such as those driven by Nrf2, should enforce tissue damage control and disease tolerance to systemic infections [12] (Figure 2). Presumably, this occurs via the expression of Nrf2 regulated effector genes, such as those controlling glutathione synthesis/conjugation [13,18], haeme metabolism, i.e. HO-1 [56–58], iron metabolism, e.g. FtH [59,60], ferroportin-1 [31] and/or lipid peroxidation, e.g. biliverdin reductase [61]. Other mechanisms underlying the protective effects of Nrf2 were linked to maintenance of mitochondrial function [51].

## Trade-off of the stress response driven by Nrf2

Disease tolerance mechanisms do not exert a negative impact on pathogens. As such, stress responses underlying disease tolerance create a situation in which the infected host, although healthy, can transmit the disease. This has probably major consequences on the natural selection of genes regulating stress responses, including Nrf2 [62]. Moreover, stress responses preserve core cellular functions at the expense of ‘accessory’ ones [63–65] and therefore must be tightly regulated over time [11]. Nrf2 is no exception to this rule as illustrated by the observation that chronic Nrf2 activation promotes tumorigenesis [66].

## Conclusion

The stress response regulated by Nrf2 probably plays a major role in conferring disease tolerance to systemic infections, such as those triggered by bacteria infection and leading to severe sepsis or the one triggered by *Plasmodium* infection and leading to severe forms of malaria. Viral infections, on the other hand, appear to thrive on host Nrf2 activation, as illustrated by a number of examples in which induction of Nrf2 activity favours virus proliferation. Given the above, it is not clear to what extent the Nrf2 signal transduction pathway may be targeted to treat infectious diseases.

## Abbreviations

ATF3: activating transcription factor 3
Cul: cullin
FtH: Ferritin H chain
GSK: glycogen synthase kinase
Hrd1: HMG-coA reductase degradation 1
IL: interleukin
iNOS/NOS2: inducible form of nitric oxide synthase
Keap1: Kelch-like ECH-associated protein 1
LPS: lipopolysaccharide
Mø: macrophages
NF-κB: nuclear factor kappa B
Nrf2: nuclear factor-erythroid 2-related factor 2
ONNO^−^: peroxinitrate
Rbx: RING box protein
RNS: reactive nitrogen species
ROS: reactive oxygen species
SCF: Skp1–Cul1–F-box
SCF^β-TrCP^: SCF–β-TrCP complex
sMaf: small musculoaponeurotic fibrosarcoma
TLR4: toll-like receptor 4
β-TrCP: β-transducin repeats-containing protein
